# Supplementary material for: Clinical leaders crossing boundaries: A study on the role of clinical leadership in crossing boundaries between specialties
Source: PLoS One. 2023 Nov 9;18(11):e0294264. doi: 10.1371/journal.pone.0294264 (PMC10635562; doi:10.1371/journal.pone.0294264)
Supplement: S1 Table — (DOCX) [file pone.0294264.s001.docx]

**S1 Table. Survey questions and answer options.**

|  | **Questions** | **Answer options** |
| --- | --- | --- |
| **Gender** |  | female; male; (non)-binary; rather not say |
| **Function** | What is your function? Please give the answer that best describes your function | medical specialist; junior doctor; junior doctor in training; rather not say |
| **Specialty** | I am part of the medical specialty group | Allergology; anaesthesiology; cardiology; surgery; dermatology; intensive care; rehabilitation medicine; urology; internal medicine; geriatrics; oral surgery; ENT; paediatrics; gynaecology; pulmonary medicine; neurology; ophthalmology; emergency medicine; nuclear medicine; psychiatry; orthopaedics; pathology; rheumatology; gastrointestinal; plastic surgery; radiology; radiotherapy; other. |
| **Formal leadership position** | 1. I am coordinator of the medical specialty.  2. I am manager of an accountable multidisciplinary thematic unit | yes; no |
| **Tenure** | 1. How long have you worked in this hospital?  2. How long have you been working in your current profession? | less than 1 year; 1 to 5 years; 6 to 10 years; 11 to 15 years; 16 to 20 years; more than 21 years; rather not say |
| **Clinical Leadership** | 1. When I am concerned about the patient’s wellbeing, I take risks by questioning orders and/or treatments.  2. I am able to provide evidence-based rationale for my clinical decisions.  3. I engage in reflective practice and try to understand what went well and what did not go well  4. I negotiate with and support members of the interprofessional health care team to help patients achieve their goals.  5. I am engaged when communicating with patients to achieve patient-centred goals.  6. I engage in meaningful conversations with colleagues to foster our ability to provide patient-centred care.  7. I actively listen to colleagues’ diverse points of view.  8. I establish therapeutic relationships with patients and their families that are based on trust.  9. I develop cooperative relationships with my peers and colleagues  10. I do my best to follow through on the promises and commitments I make to patients  11. I try to ensure we work toward achievable goals and establish measurable objectives in achieving clinical patient outcomes.  12. I am committed to patient centred care.  13. I publicly acknowledge my colleagues who exemplify commitment to professional values.  14. I provide positive feedback to colleagues when their actions contribute to the wellbeing of patients and their families.  15. I find ways to celebrate colleagues’ accomplishments. | almost never; occasionally; some of the time; most of the time; almost always |
| **Relational Coordination** | The following questions are about collaboration with other medical specialists (a. specialists from the own medical specialty, b. with specialists from different specialty groups (working in our study hospital in the same thematic unit)). Please let us know which option you believe to be most suited.  1. How frequently do you communicate with…?  2. Do … with you in a timely manner?  3. Do … with you accurately?  4. When problems arise regarding care, do … work with you to solve the problem?  5. Do … know the work you do in care?  6. Do … respect the work you do in care?  7. Do … share your goals? | never; rarely; occasionally; mostly; all the time |
| **Quality of Care** | Overall, how would you describe the quality of patient care within your medical specialty department? | poor; fair; good; excellent |
| **Job Satisfaction** | How satisfied are you with your current job in this hospital, on a scale of 0 to 100? | 0 - 100 |
