# Supplementary material for: Clinical leaders crossing boundaries: A study on the role of clinical leadership in crossing boundaries between specialties
Source: PLoS One. 2023 Nov 9;18(11):e0294264. doi: 10.1371/journal.pone.0294264 (PMC10635562; doi:10.1371/journal.pone.0294264)
Supplement: S2 Table — (DOCX) [file pone.0294264.s002.docx]

**S2 Table.** Mean scores and differences between physicians in a formal leadership position compared to those not in a formal leadership position.

| Variable | Formal Leader | n | Mean | SD | *t* | df | *p* |
| --- | --- | --- | --- | --- | --- | --- | --- |
| Clinical Leadership | Yes  No | 23  77 | 3.92  4.05 | 0.11  0.04 | 1.346 | 98 | .181 |
| Relational Coordination:  Physicians from same specialty group | Yes  No | 21  73 | 4.37  4.42 | 0.12  0.06 | 0.376 | 92 | .708 |
| Relational Coordination:  Physicians from different specialties | Yes  No | 23  80 | 3.74  3.89 | 0.14  0.06 | 1.174 | 101 | .243 |
| Quality of Care | Yes  No | 23  80 | 3.30  3.35 | 0.10  0.06 | 0.348 | 101 | .729 |
| Job Satisfaction | Yes  No | 23  80 | 79.65  80.85 | 3.25  1.37 | 0.387 | 101 | .699 |
