# Supplementary material for: Clinical leaders crossing boundaries: A study on the role of clinical leadership in crossing boundaries between specialties
Source: PLoS One. 2023 Nov 9;18(11):e0294264. doi: 10.1371/journal.pone.0294264 (PMC10635562; doi:10.1371/journal.pone.0294264)
Supplement: S3 Table — (DOCX) [file pone.0294264.s003.docx]

**S3 Table.** Correlations matrix for relational coordination items with job satisfaction and quality of care.

| **Item** | **2** | **3** | **4** | **5** | **6** | **7** | **8** | **9** |
| --- | --- | --- | --- | --- | --- | --- | --- | --- |
| Relational Coordination dimensions |  |  |  |  |  |  |  |  |
| *High quality communication* |  |  |  |  |  |  |  |  |
| 1. Frequent | .60*** | .44*** | .58*** | .62*** | .61*** | .57*** | .38*** | .41*** |
| 2. Timely |  | .63*** | .64*** | .53*** | .61*** | .56*** | .20* | .38*** |
| 3. Accurate |  |  | .64*** | .49*** | .56*** | .52*** | .27** | .43*** |
| 4. Problem-solving |  |  | . | .69*** | .73*** | .60*** | .27** | .51*** |
| *High quality relationships* |  |  |  |  |  |  |  |  |
| 5. Shared goals |  |  |  |  | .68*** | .63*** | .33*** | .55*** |
| 6. Shared knowledge |  |  |  |  |  | .66*** | .40*** | .50*** |
| 7. Mutual respect |  |  |  |  |  |  | .44*** | .50*** |
| *Outcomes* |  |  |  |  |  |  |  |  |
| 8. Quality of Care |  |  |  |  |  |  |  |  |
| 9. Job Satisfaction |  |  |  |  |  |  |  |  |
| Significance: * *p* <.05, ** *p <.01, *** p <.001* | | | | | | | | |
