# Supplementary material for: Clinical leaders crossing boundaries: A study on the role of clinical leadership in crossing boundaries between specialties
Source: PLoS One. 2023 Nov 9;18(11):e0294264. doi: 10.1371/journal.pone.0294264 (PMC10635562; doi:10.1371/journal.pone.0294264)
Supplement: Attachment — Submitted filename: Response to Reviewers.docx [file pone.0294264.s005.docx]

Dear Philip A. Cola,

On behalf of my coauthors and myself, many thanks for the opportunity to revise our manuscript PONE-D-23-06594, entitled “Clinical leaders crossing boundaries: a study on the role of clinical leadership in crossing boundaries between specialties”. We thank the reviewers and the editor from PLOS ONE for taking the time to examine our manuscript. We are pleased to hear the interest in our study. The suggestions and remarks were very helpful to improve our manuscript. We have taken great care in responding to all the comments of the reviewers and have provided a detailed response to each of the concerns raised.

We have taken the liberty of numbering points raised by reviewers and have presented our response after each comment. Corresponding changes in the revised manuscript have been tracked for easy recognition.

We hope that the revised manuscript satisfactorily addresses the noted comments. Thank you again and we look forward to hearing from you.

Yours sincerely,

On behalf of all authors,

Anoek Braam

**Comments from the Academic Editor.**

**1. We are very interested to have reviewed your important study around relational coordination and boundary spanning across clinical leaders.  We believe that improved collaboration and transdisciplinary clinical environments will benefit patients and care givers alike.  However, a main aim of the study around the relational coordination and patient outcomes does not seem to be addressed clearly in the outcomes of the paper.  Furthermore, there does not appear to be direct empirical evidence of meaningful patient outcomes related especially to those with co-morbidities that improve based on the data from the relational coordination scores.  It seems that perhaps the data needs to be analyzed a bit more in depth to answer directly these aims or goals of this study.  It is believed that the data is available in this sample, but not analyzed perhaps in depth enough to answer the main questions directly.**

The aim of our study as presented in the manuscript is indeed **to explore the associations between clinical leadership, relational coordination, and outcomes.** However, we now realize that the use of the word outcomes may be confusing. We refer to the outcomes as discussed in the manuscript, namely job satisfaction and quality of care and **not** patient outcomes. We **do not** have data on patient outcomes and are also unable to obtain them at this time. Therefore, we are only able to address your feedback by re-writing the manuscript in a way that the type of outcome measures we collected is made clearer. For example, by reframing the aim of our study in the following: “*In this study, we aim to explore the associations between clinical leadership, relational coordination, and outcomes in terms of job satisfaction and physicians reported quality of care*.” And introducing hypotheses 2a and 2b as follows: ‘*Therefore, we propose the following regarding the relationship between relational coordination among physicians and physicians reported quality of care and job satisfaction:’.*

After processing the comments of the other reviewers, we believe the current version of the manuscript is clearer and provides, as mentioned by reviewer 1 “*an important insight into the importance of relational coordination in the hospital setting, in particular an important correlation between relational coordination and several important qualities contributing to successful medical care, including care quality, leadership, and job satisfaction both within and between specialties.”*

**2**. **Additionally, some participants are leaders and others are not leaders (at least not yet) and therefore this mixed sample could potentially introduce bias into the results that have been provided or that we are now currently asking for more details on.  Please address the potential for bias as all participants are not at leadership positions currently.**

We acknowledge the concern regarding bias created by whether or not a physician has a formal leadership position as brought up by the editor. Encouraged by comments from the editor as well as one of the reviewers we have tested (using independent samples t-tests) whether there were statistical differences in scores used in the analyses for respondents with/without a formal leadership position. These analyses showed no statistically significant differences in clinical leadership, relational coordination, job-satisfaction, and quality of care scores for these two groups.

Considering that these analyses contribute to the robustness of the study, we briefly touch upon these analyses as a robustness check. We have added the following section in the manuscript: ‘***Additional analyses.*** *Independent sample t-tests were performed to compare scores from physicians in a formal leadership position with those who are not, to assess the robustness of the study’s findings (see Appendix X). The independent t-tests showed no differences between physicians’ clinical leadership scores (t (98) = 1.35, p = .18 two tailed) for physicians in a formal leadership role (M = 4.05, SD = .38) compared to those not in a formal leadership role (M = 3.92, SD = .50), nor for the other variables used in the analyses.’*

**Comments from Reviewer #1**

We would like to thank reviewer 1 for the carefully considered suggestions. We have tried to incorporate each suggestion as best we can.

**Major Points:**

**3. The associations between relational coordination and patient outcomes, as stated as the aim of the study, are not addressed. There is no evidence provided of tangible or other reported patient outcomes (particularly, patients with co-morbidities) in different thematic units is correlated to relational coordination score, and the score of physician assessment of their own quality care introduces a major bias despite “proven validity”. Several participants also had formal leadership positions, which may further bias the study results. To prove this claim, the authors should ask question of care satisfaction to their target population – patients with comorbidities – and correlate with the thematic unit responsible for care, if possible.**

The aim of our study as presented in the manuscript is indeed **to explore the associations between clinical leadership, relational coordination, and outcomes.** The use of the word outcomes may be confusing; we here refer to the outcomes as discussed in the manuscript, namely job satisfaction and quality of care and **not patient outcomes.** Although we agree that patient outcomes would have added value, we **do not** have data on patient outcomes and are also unable to obtain them at this time. To see how we tried to clarify the outcomes used, please see point 1.

We agree with the reviewer that the use of physicians’ own assessment (self-reporting) of quality of care introduces bias. Therefore, we have mentioned this in the limitations section of our research. *‘Third, we used self-reported measures that, despite the guarantee of respondents’ anonymity, are subject to various biased, such as social desirability and common method and source bias.’.*

We acknowledge the concern raised by reviewer 1 and the editor about bias arising from whether or not a physician has a formal leadership position. Please, we refer to point 2 to see how we have dealt with this suggestion.

**4. It would be helpful to have a more in-depth analysis of the correlation of individual questions and points with outcomes, not just as grouped under “relational coordination.” For example, how do the individual components of communication (frequency, timeliness, etc.) correlate with quality of care?**

Based on the reviewers’ suggestion and in line with earlier research^[1]^ we have conducted and added a more in-depth analysis of the correlation of individual items of relational coordination and outcomes in terms of job satisfaction and quality of care. These additional analyses did not lead to any insights other than those presented in our manuscript.

Considering that these analyses contribute to the robustness of the study, we briefly touch upon these analyses and put the table in the appendix. We added the following in the manuscript: *‘To assess the robustness of the study’s findings, we also conducted separate correlation analyses for the items of relational coordination (i.e., frequent, timely, accurate, problem-solving, shared goals, shared knowledge, mutual respect) with job satisfaction and quality of care. The results from these analyses (see Appendix X) were equivalent to those from the presented main analyses.’*

**5. Additionally, a differentiation of how responders that hold formal leadership positions responses differ from those that do not could be informative to support several claims in the manuscript.**

Based on this and the suggestion of the academic editor we have tested whether there were statistical differences between respondents with/without a formal leadership position. We found that these two groups did not show statistically significant differences in clinical leadership, relational coordination, job-satisfaction and/or quality of care scores. We refer to point 2 to show how we have incorporated this into the manuscript. Our claims are focused on informal clinical leadership roles.

**6. A more detailed explanation of how to read Table 1 should be provided in the legend to assist with interpretation.**

Based on this comment we have looked at other PLOSONE publications (for example in 2019^[2]^) to improve the legend of table 1. We have now added additional information to help the reader with interpretation of the table. Based on articles published in *PLOSONE* we have adjusted the table legend: *Significance: * p <.05, ** p <.01, *** p <.001; Strength: .10 to .29 is weak; .30 to .49 is moderate, .50 to 1.00 is strong.*

**7. The analysis of the stronger association with the outcome of job satisfaction and relational coordination seems to be biased considering the strong associations between other measured items (i.e. between clinical leadership (group 1) and relational coordination (group 3)) and that these are different based on relational coordination from same specialty group or different specialty groups. More thought should be placed on how those high Pearson product-moment correlations impact the study.**

We have now put more emphasis on the stronger association between relational coordination and job satisfaction/quality of care for physicians from the same specialty group compared to physicians from different specialty groups. Therefore, we have revised the end of the *correlations* section in the results, that now reads: “*Compared to relational coordination between physicians from different specializations, there is a stronger correlation between relational coordination between physicians from the same specialist group and job satisfaction and quality of care. However, relational coordination in all its forms shows positive correlations with job satisfaction and quality of care.’*

We believe there is no reason to assume multicollinearity as the independent variable are not highly correlated (see Table 1). According to Julie Pallant^[3]^ multicollinearity only exists when the independent variables are highly correlated (*r =* .7 and above).

**8. The authors conclude that clinicians should strive to be clinical leaders, which goes hand in hand with job satisfaction. However, there does not seem to be a metric about clinician desire to be an official leader (e.g., have a leadership role in the future, head a department, etc) and how that correlates with relational coordination scores. This would be an important data point to support that claim.**

The focus of our study is on the informal role of clinical leadership not on formal leadership roles. We do not have a metric about clinicians’ desire to be an official leader, but we have now addressed formal versus informal leadership see point 2 and 5. Our claim that physicians should strive to be clinical leaders, which goes hand in hand with job satisfaction is based on the results of our sequential mediation analyses in which we state the following *“A significant total effect of clinical leadership on job satisfaction was found, indicating that more clinical leadership was associated with greater job satisfaction when the mediators were not taken into account.”*

**9. If possible, a supplementary table of questions that were provided to participants should be provided.**

We have added a supplementary table with the questions provided to the participants. We refer to the appendix in the *methods* as follows: *‘From October to December 2020, we conducted a cross-sectional survey (see appendix X) among physicians.*

**10. Some conclusions made in the manuscript are not entirely supported by the data. For example, the conclusion that this study shows clinical leaders that demonstrate behaviors such as enabling others to act and sharing an inspiring vision will most likely contribute to the improvement strategies is not proven with the data provided. More thought should be given to claims made throughout the text, and they should be modified based off of what the results of the survey support.**

After re-reading the conclusion mentioned by the reviewer, we acknowledge that this is unclear to the reader. Enabling others to act and sharing an inspiring vision are sub-scales of the used clinical leadership scale, which were named in the method, but not further addressed in the analyses. We believe the real concern lies in mentioning *contribute to these intergroup-relation-improvement strategies.*

We have replaced this conclusion to now read: “*Our research demonstrates that self-perceived clinical leaders who exhibit behaviors like having deep dialogues with peers are more inclined to collaborate with physicians from other specialties. Clinical leaders appear to help strengthen intergroup relations.”*

We have thoroughly reviewed the discussion section. After processing the other comments, we believe the current conclusions are based on the results of the survey.

**Minor Points:**

**11. Several hypotheses are provided in the introduction of the manuscript. The study might benefit from consolidating hypotheses into one over-arching hypotheses for clarity and for conciseness.**

We implemented the reviewer’s suggestion by adding a sentence after the aim of the study which shows an over-arching expectation. *“Where we anticipate relational coordination and clinical will both positively influence outcomes, with relational coordination acting as a mediator between clinical leadership and outcomes.”*

**12. The main manuscript text can get repetitive, especially in the background section. It could benefit from revision to make points more concise.**

Upon the reviewer’s suggestion, we conducted a thorough re-read of the background section. We have revised sentences and believe that we have thereby reduced the repetitive nature of the text. Revisions are tracked in the manuscript with track changes. As an example: ***OLD*** *“This is why relational coordination, especially in times when physicians encounter the challenge of improving quality outcomes while also responding to cost pressures seems to be a promising mechanism for improving quality of care”* ***NEW*** *“This is why relational coordination appears to be a promising mechanism for raising the standard of care while also addressing financial pressures.”*

**13. The discussion could be expanded to include future directions and additional areas where this survey could be applicable.**

We have added the following to our limitations section: *‘To further understand the collaboration between physicians of various specialties and care coordination, it would be beneficial to conduct similar studies in other (types of) hospitals as the study was only conducted in one. In addition, other outcome metrics, such as patient outcomes, may also contribute to a deeper comprehension. Finally, the current study only used relational coordination between relatively large groups, potentially important insights could be gained by looking at collaboration between physicians per specialty.’.*

**Comments from Reviewer #2**

We also like to thanks reviewer 2 for the clear and helpful suggestions, which we all tried to incorporate.

**Background:
14. Line 72. Please add a brief definition of clinical leadership or strong clinical leadership**

The word “strong” was deleted. While the following sentence was added as brief definition of clinical leadership: “Clinical leaders are physicians who from an informal position take the initiative to contribute to and encourage others to improve care.”

**15. Line 85-86. Please provide an example or explanation as to why it is more difficult for physicians from different medical specialties to effectively coordinate care**

After rereading these sentences thoughtfully, we believe the examples are already given prior to the sentence in question. Due to the chosen words and sentence-structure, this link was not sufficiently clear. This has been changed to now read: ‘Historically, less focus was on facilitating these conditions for physicians from different medical specialties.” In which *these conditions* are referring to the conditions described in earlier sentences of the paragraph.

**16. Line 90-91. This information is repeated in the previous paragraph, please revise**

The following part “which reviewed all empirical studies on predictors and outcomes of relational coordination published from 1991 to 2019” was deleted. It now reads: “The review by Bolton, Logan, and Gittell, also provides evidence that … and staff outcomes (e.g., job satisfaction, lower burnout rates).”

**17. Line118 consider choosing just one term: physician versus doctor.**

We revised the term as suggested by the reviewer.

**18. Line 119. Please add an example following this sentence “Although doctors lead from the day they start clinical practice, crossing medical specialist boundaries might be more demanding”**

We have revised the mentioned sentence and added an example: ‘*Although, physicians are used to play such roles within their specific specialist setting, they are now expected to assume responsibilities across disciplines, crossing medical specialist boundaries. However, research seems to suggest there are considerable barriers for physicians to take on such roles such as poor interdisciplinary relationships, role conflict, and resistance to change.*’

**19. Line 131-133. Suggest moving “a” to precede the first instance of “relational coordination” in the hypothesis so that is reads: Hypothesis 3: Clinical leadership behaviors are positively related to (a) relational coordination among physicians within their medical specialty group and (b) relational coordination among physicians from different medical specialties.**

We revised the sentence to the sentence suggested by the reviewer.

**Methods
20. Line 252. Change ‘respondents’ to participants’**

We revised the term as suggested by the reviewer.

**21. Line 270-271. Change the sentence to read: Participants first agreed to participate in the study before data collection and their identities are kept confidential.**

We revised the sentence to the sentence suggested by the reviewer.

**Discussion:
22. Line 374. please provide elaboration on Amy Edmondson’s work by describing a teaming application appropriate for physician practice and multimorbid care collaboration**

Based on the suggestion of the reviewer we have revised the sentence on Amy Edmondson’s work on teaming. To now read: “*The work by Amy Edmondson on teaming provides an interesting alternative perspective stating that organizational culture and physicians’ mindsets need to be reframed; creating awareness among physicians on how their own expertise interacts with other specialties. With the goal of creating fluid, collaborative, interdependent multidisciplinary teams based on patients’ needs with a shifting mix of partners across organizational boundaries*.”

**23. Line 387. insert ‘as’ between “such social” to read “such as social desirability”**

We have inserted the word as suggested by the reviewer.

**24. Line 390. Suggest adding “ and care coordination” at the end of the sentence.**

We have added to the sentence as suggested by the reviewer.

**25. Practical Implications. As in the abstract, it seems odd to justify the need for specialty group practice. Perhaps adding that more structure/intervention/purposeful socialization is needed between areas of specialty practice in order to take advantage of expertise toward patients’ benefit.**

**Abstract: Implications for practice seems a bit odd as the research does not aim to justify continued specialty practice teams. Please consider an implication more closely tied to the importance of promoting relational coordination among physicians and how this might consistently and systematically promoted.**

Upon reviewing the practice implications again, we agree with the reviewer that it is seems odd justifying the need for specialty group practice and the implications could benefit from focusing more on the importance of relational coordination among physicians. Within the abstract it now reads: ‘*To improve multidisciplinary collaboration, managers should encourage clinical leadership and pay attention to the strong relationships between physicians from the same specialty.*’.

We have completely revised the practical implications section, it now reads: ‘*First, our findings suggest that physicians should strive to demonstrate clinical leadership behaviors, as these are associated with increased job satisfaction. In addition, managers should encourage clinical leadership by physicians because the behaviors they exhibit foster relationships among physicians and can strengthen interdisciplinary collaboration. Second, as seen in this study, there is still potential for a further increase in relational coordination between physicians from different specialties compared to those between physicians from the same specialty. Only the introduction of multidisciplinary structures (as implemented in the study hospital), may not (yet) offer sufficient support to fully commit to multidisciplinary care. As a first step towards future improvement of the quality of collaboration, managers could discuss levels of relational coordination amongst members of the multidisciplinary unit. In addition, focus on multidisciplinary care should be embedded in, amongst others, training and medical quality review, to encourage collaboration and reduce focus on specialist silos. Third, in contrast to earlier suggestions, our findings show that currently the medical specialty group is like a bird’s nest. It provides physicians with a stable base which helps them to explore and form multidisciplinary collaborations. So, when encouraging multidisciplinary collaboration between physicians’ focus should not only be on the multidisciplinary relations, but they should also continue to pay attention to strong connections between physicians from the same specialty.’*

1. Havens, Vasey, Gittell, & Lin (2010). Relational coordination among nurses and other providers: impact on the quality of patient care. *Journal of Nursing Management.*
2. Czekierda, Zarychta, Knoll, Keller, & Luszcyznska, 2019. Links between meaning in life and physical quality of life after rehabilitation: mediating effects of positive experiences with physical exercises and mobility. doi: 10.1371/journal.pone.0224503
3. Pallant. 2020. SPSS Survival Manual, a step-by-step guide to data analysis using IBM SPSS. 7^th^ edition. Mc Graw Will, Open University Press.
